# Dielectrophoretic Capture and Genetic Analysis of Single Neuroblastoma Tumor Cells

**DOI:** 10.3389/fonc.2014.00201

**Published:** 2014-07-31

**Authors:** Erica L. Carpenter, JulieAnn Rader, Jacob Ruden, Eric F. Rappaport, Kristen N. Hunter, Paul L. Hallberg, Kate Krytska, Peter J. O’Dwyer, Yael P. Mosse

**Affiliations:** ^1^Division of Hematology and Oncology, Perelman School of Medicine at the University of Pennsylvania, Philadelphia, PA, USA; ^2^Division of Oncology, Center for Childhood Cancer Research, Children’s Hospital of Philadelphia, Philadelphia, PA, USA; ^3^Department of Pathology and Laboratory Medicine, Perelman School of Medicine at the University of Pennsylvania, Philadelphia, PA, USA; ^4^Department of Pediatrics, Perelman School of Medicine at the University of Pennsylvania, Philadelphia, PA, USA

**Keywords:** neuroblastoma, disseminated tumor cell, circulating tumor cell, single cell capture, whole genome amplification, targeted sequencing

## Abstract

Our understanding of the diversity of cells that escape the primary tumor and seed micrometastases remains rudimentary, and approaches for studying circulating and disseminated tumor cells have been limited by low throughput and sensitivity, reliance on single parameter sorting, and a focus on enumeration rather than phenotypic and genetic characterization. Here, we utilize a highly sensitive microfluidic and dielectrophoretic approach for the isolation and genetic analysis of individual tumor cells. We employed fluorescence labeling to isolate 208 single cells from spiking experiments conducted with 11 cell lines, including 8 neuroblastoma cell lines, and achieved a capture sensitivity of 1 tumor cell per 10^6^ white blood cells (WBCs). Sample fixation or freezing had no detectable effect on cell capture. Point mutations were accurately detected in the whole genome amplification product of captured single tumor cells but not in negative control WBCs. We applied this approach to capture 144 single tumor cells from 10 bone marrow samples of patients suffering from neuroblastoma. In this pediatric malignancy, high-risk patients often exhibit wide-spread hematogenous metastasis, but access to primary tumor can be difficult or impossible. Here, we used flow-based sorting to pre-enrich samples with tumor involvement below 0.02%. For all patients for whom a mutation in the Anaplastic Lymphoma Kinase gene had already been detected in their primary tumor, the same mutation was detected in single cells from their marrow. These findings demonstrate a novel, non-invasive, and adaptable method for the capture and genetic analysis of single tumor cells from cancer patients.

## Introduction

Metastatic cancer is almost uniformly lethal and occurs when circulating or disseminated tumor cells (CTC/DTCs) engraft sites distant from the primary tumor (1). Metastatic disease remains a challenge not only to treat but also to study, as tumor samples in patients with advanced cancer are often difficult to obtain. Lack of surgical access to tumor in this setting implies that prognostic and treatment decisions must be based on primary tumor attributes, often from a single time point, and in the absence of a characterization of metastatic cells, despite known genomic variation between CTC/DTCs and primary tumor (2–4). Indeed, recent studies of prostate (5), breast (6), and other cancers (7, 8) suggest considerable cell to cell genomic heterogeneity within different tumor biopsies from an individual patient, challenging the efforts to monitor solid cancer heterogeneity. While CTCs from the blood are often studied, tumor cells, which have disseminated to the marrow (DTCs), may also form a reservoir of cells that could seed distant metastases (9), and are less well characterized. In addition, the advent of newer targeted therapies mandates molecular assessment of both the primary and circulating/disseminated tumor to comprehensively determine target molecule expression. Thus, accessing the circulating and disseminated tumor compartments via serial blood draws and/or bone marrow biopsies provides a non-invasive approach for studying the implications of heterogeneity, and fully realizing the benefits of targeted therapies. Moreover, CTC/DTCs can theoretically be assayed as markers of minimal residual disease, and, once isolated, can be probed for molecular diagnostic and/or therapeutic response information. Gaining fundamental knowledge about the cell to cell diversity of cancer metastasis will be critical for understanding if therapeutic targets identified in primary tumor tissue are relevant in the metastatic cells, and a key driver for the development of rational and effective early phase clinical trials for cancer patients.

Single cell capture has generally relied on labor-intensive, low throughput techniques such as manual micromanipulation of EpCAM- or cytokeratin-stained cells (3, 10) or *in situ* hybridization-based analysis of tissue sections (11, 12). More recent studies, however, hint at the wealth of clinically relevant information to be gleaned from a more sensitive and higher throughput approach to single cell analysis (6, 12, 13). However, even with the use of technologies such as the FDA-approved CellSearch system, the detection of tumor cells in the blood or marrow of patients has often been limited to bulk analysis of EpCAM-positive tumor cells (14–17). While enumeration of these cells can provide valuable prognostic information, genetic profiling of CTC/DTCs can likely inform individualized treatment decisions and guide selection of targeted therapies. To address this, we have adopted the DEPArray microelectronics and microfluidics technology for individual tumor cell capture from pediatric bone marrow samples. This platform, recently shown to be effective for the isolation of tumor cells from lung and breast cancer patient blood samples (18, 19), utilizes dielectrophoresis (DEP) to electronically trap and move individual cells, thereby providing a means to isolate rare cells from heterogeneous samples for single cell analysis (20–22). Fluorescence-labeled cells are isolated from complex biological samples based on expression of single or multiple antigens that distinguish between tumor and cells of hematopoietic origin, thus allowing for the capture of non-epithelial tumors as well as EpCAM-negative tumor cells of epithelial origin which have undergone epithelial to mesenchymal transition (EMT).

To demonstrate the feasibility of a DEPArray-based approach to DTC isolation and genetic analysis, we have focused on neuroblastoma, a childhood malignancy of the developing sympathetic nervous system. Neuroblastoma patients present with wide-spread hematogenous based metastases in over 50% of cases (23), and tumor cells have been detected by immunocytologic approaches in the marrow of 81% and the blood of 58% of stage 4 neuroblastoma patients at diagnosis (24). Notable for its phenotypic variability and widely divergent clinical courses, the disease accounts for a disproportionate amount of childhood cancer morbidity and mortality (25). Multiple groups have used RT-PCR-based detection of neuroblastoma specific transcripts to further demonstrate that neuroblastoma is a systemic disease, and outcome is highly correlated with circulating tumor burden, and/or failure to clear disseminated cells (26–30). Recently introduced targeted therapies for neuroblastoma patients include the small molecule inhibitor Crizotinib, which targets the receptor tyrosine kinase Anaplastic Lymphoma Kinase (ALK) and was well tolerated in a recent Phase 1 dose-escalation trial (31). A randomized clinical trial of an immunotherapeutic regimen, including the ch14.18 monoclonal antibody targeting the disialoganglioside GD2, resulted in a dramatic increase in event-free survival from 46 to 66% (32). However, despite these recent advances, most high-risk neuroblastoma patients die from their disease (23). Therefore, the frequency of CTC/DTCs, the lack of EpCAM expression, the advent of targeted therapies, and the urgent need for additional therapeutic options for high risk patients make neuroblastoma an ideal disease in which to establish proof of principle for the capture and molecular analysis of DTCs. In this study, we used cell line spiking experiments to establish the sensitivity and specificity of single cell isolation and targeted sequencing, and then applied this approach to neuroblastoma patient bone marrow samples.

## Materials and Methods

### Patient samples and mononuclear cell isolation

Six de-identified neuroblastoma patient bone marrow samples were obtained from subjects enrolled on Children’s Oncology Group trial ADVL0912 (NCT00939770) (31). Written informed consent from parents or guardians was obtained, and the institutional review boards of participating institutions approved the protocol. Four additional de-identified and discarded bone marrow samples were obtained from the Children’s Hospital of Philadelphia; the use of these de-identified samples is not human subjects’ research according to our Institutional Review Board, thereby waiving the need for consent. Peripheral blood for spiking experiments was obtained from normal donors after obtaining written informed consent and using protocols approved by the Institutional Review Board of the Hospital at the University of Pennsylvania. Buffy coats from bone marrow were enriched by Ficoll centrifugation (GE Healthcare Life Sciences, Piscataway, NJ, USA). If cells were not immediately used for additional assays, fixation was performed using Miltenyi Inside Fix (Miltenyi Biotec Inc., Auburn, CA, USA).

### Cell lines and spiking experiments

**Figure 1:**
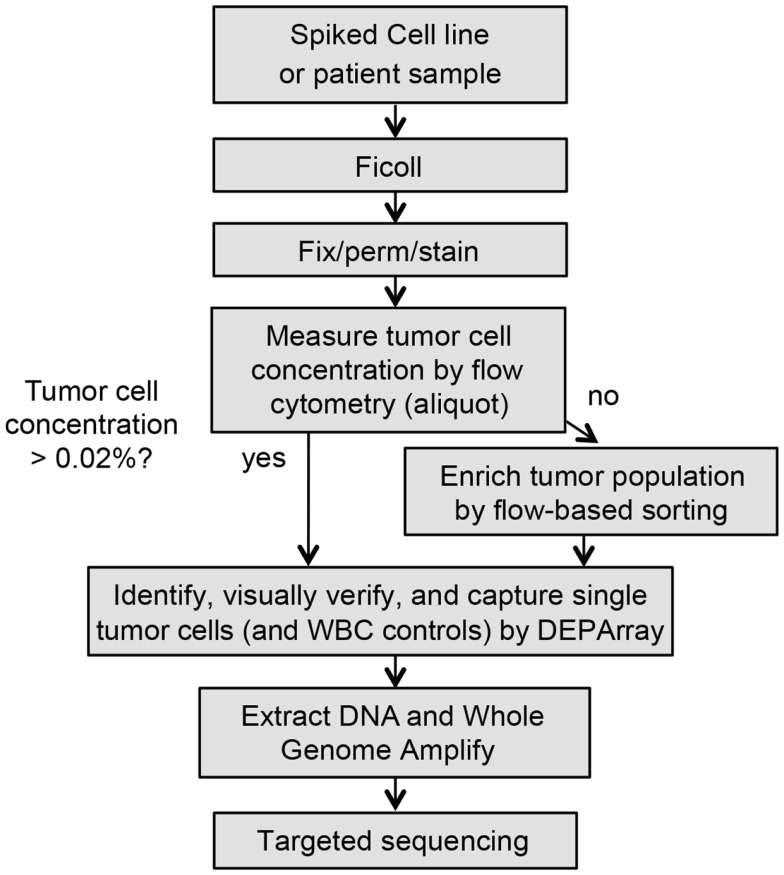
**Workflow for rare single cell isolation by DEPArray**. Shown are the steps for dielectrophoretic isolation and downstream genetic analysis of single cells. Because the DEPArray cartridge has a capacity of ~40,000 cells, if the tumor cell concentration is below 0.02%, pre-enrichment of the sample is first completed on the FACSAria.

All cell lines were obtained from the Children’s Hospital of Philadelphia cell line bank. The NB1643M cell line is a sub-clone of NB1643, which is homozygous for the known ALK R1275Q mutation. All cell lines were maintained in a 5% CO_2_ incubator at 37°C in complete RPMI (Invitrogen, Carlsbad, CA, USA). Tumor cell identity was authenticated using AmpFLSTR Identifiler (Applied Biosystems, Grand Island, NY, USA) and all cell lines were routinely mycoplasma tested. Adherent cell lines were grown to 70–80% confluency before being used for spiking experiments, then trypsin-treated for dissociation from the flask surface. For spiking experiments, cell lines and white blood cells (WBCs) were washed twice separately and counted. Cell lines were then serially diluted into WBCs at the indicated ratios ranging from a tumor:WBC ratio of 1:10 to 1:1,000,000. Spiked samples were then fluorescently labeled and entered into the DEPArray (Silicon Biosystems, Bologna, Italy) workflow as described below and depicted in Figure 1. Due to the 40,000 cell capacity of the DEPArray cartridge, pre-enrichment of the sample was first completed on the FACSAria II (Becton Dickinson Franklin Lakes, NJ, USA) prior to running on the DEPArray, if the tumor cell concentration was below 1 in 5,000 WBCs (0.02%). At this tumor cell concentration, a full cartridge containing 40,000 cells would be likely to contain only 8 tumor cells, a number below which it would be difficult to conduct meaningful downstream analysis. For most experiments described here, FACSAria-based enrichment yielded less than 40,000 total cells and, therefore, all enriched cells were injected into a single DEPArray cartridge. For the two patient samples for which the number of enriched cells exceeded 40,000, the FACSAria output was divided between two DEPArray cartridges.

### Antibodies and flow cytometry

Flow cytometry was conducted using an Attune cytometer (Life Technologies, Grand Island, NY, USA) and Venturi software (Applied Cytometry, Dinnington, UK). Cells were analyzed using isotype antibody controls, Violet/SYTOX (Life Technologies) to detect apoptotic cells, Hoechst 33342 nucleic acid stain (Life Technologies), and monoclonal antibodies obtained from BD Biosciences (BD340723 CD56-FITC, BD554272 14.18 GD2-PE, BD347198 EpCAM-PE, BD347197 EpCAM-FITC, BD555997 EGFR-PE), Invitrogen (MHCD45054 CD45-APC, MHCD45014 CD45-FITC), and BioLegend (324406 HER-2-PE). Prior to staining, all antibodies were prefiltered using Millipore (Billerica, MA, USA) 0.65 μm Ultrafree-MC centrifugal filter units.

### Isolation of single tumor cells by DEPArray

Isolation of single cells from patient and spiked samples can be a lengthy and complex process. In order to ensure that yield and quality of isolated cells are not compromised, it is recommended that the following steps be completed in as rapid succession as possible. Reagents and materials should be assembled ahead of time, and all steps were followed precisely. As depicted in the flow chart in Figure 1, whole blood or marrow samples were subjected to Ficoll (GE Healthcare Life Sciences) centrifugation, and the post-Ficoll suspension of cells processed live or, when necessary, fixed for later analysis. Cells were counted, and then stained for the relevant tumor antigen(s), as well as the negative control CD45. To determine the tumor cell frequency of unenriched patient samples, an aliquot of ~1 million total cells was analyzed by flow cytometry. The percentage of tumor cells was determined by calculating the number of cells positively expressing the tumor antigen(s) of interest but not CD45. As described above, pre-enrichment of the sample was first completed on the BD FACSAria II prior to running on the DEPArray, if the tumor cell concentration was below 0.02%. Sorts were conducted using 70 μm nozzles at 70 psi with cells collected into a conical containing phosphate buffered saline (PBS) and 2 mM ethylenediaminetetraacetic acid (EDTA). Prior to loading into the DEPArray cartridge, cells were maintained in a polypropylene conical to minimize cell loss during two subsequent washes. Cells were then resuspended in SB115 buffer (Silicon Biosystems) if fixed or complete RPMI if cells were live, and 14 μl of sample and 800 μl of buffer were loaded into the cartridge. Of the 14 μl of loaded sample, 9.26 μl (or 66% of the total volume) then filled the electrode-embedded chamber from which single cells were selected, therefore, CTC/DTC yield was never expected to approach 100%. The cartridge was then placed in the DEPArray apparatus and non-uniform electrical fields applied. The system does not require *a priori* thresholds, and, therefore, all events are displayed in the Cell Browser scatter plot, and gating applied after visual inspection of events. Individual or small groups of cells were captured into 0.2 ml microtubes (Applied Biosystems). Following collection, each cell/tube was then washed once in PBS and the supernatant carefully removed, leaving the cell(s) in approximately 1 μl of volume. Cells were then frozen at -20°C pending downstream analysis.

### Single cell whole genome amplification, PCR, and sequencing

The Ampli1 whole genome amplification (WGA) approach (Silicon Biosystems) is based on adaptor-mediated PCR following site-specific DNA digestion with all reactions performed in one tube to avoid template loss and thus resulting in a library of highly concentrated DNA fragments 0.2–2 kb in length. Given the minute amounts of DNA being amplified and the risk of sequencing results being influenced or compromised, routine lab habits and techniques are likely incompatible with the generation of reliable and accurate sequencing results from single cells. To address this, procedures are described below in considerable detail and should be followed precisely. In order to prevent carryover of amplified DNA to single cell samples, once single cells were obtained in individual microtubes by DEPArray, all downstream processing was completed in two separated rooms: one dedicated to WGA and the other to PCR. Each room contained its own PCR hood, pipettes, racks, Veriti thermal cycler (LifeTech), freezer, and centrifuge (VWR, Radnor, PA, USA). Access to these areas was only permitted for single cell work and single-use gowns were worn rather than lab coats to reduce the chance of cross-contamination. After each experiment, all work areas were wiped down with DNA AWAY (Molecular BioProducts, San Diego, CA, USA), followed by 70% ethanol, and irradiated with UV light for at least 30 min using standalone UV lamps (UVP). The Ampli1 WGA protocol was used exactly as specified by the manufacturer. In brief, lysis reaction mix was added to each sample and incubated at 42°C overnight. Next, digestion reaction mix was added to the same tube and incubated at 37°C for 3 h. Next, ligation reaction mix was added to the same tube and incubated at 15°C overnight. Finally, primary PCR reaction mix was added and the sample subjected to 45 cycles of amplification. To minimize the chance of inadvertently removing the single cell, each liquid was delivered by careful pipetting onto the wall of the tube above the other liquid already present but without touching it. Controls for all experiments included a no-cell tube containing only Ampli1 water, and unamplified bulk DNA as a positive control. The bulk DNA was always processed as the last sample to avoid cross-contamination. To determine the concentration of DNA in the WGA product of captured single cells, we used Picogreen (Life Technologies) direct fluorescent staining according to the manufacturer’s directions. In brief, serial dilutions of the lambda DNA stock provided in the kit were first used to create a standard curve. A 2 μl aliquot of sample DNA was then added to 195 μl of a 1:200 dilution of Picogreen reagent in TE for a final volume of 197 μl. Fluorescence was measured on a GloMax Luminometer (Promega, Madison, WI, USA) at emission/excitation wavelengths of 490/520, and sample concentrations were calculated based on the standard curve. One microliter of WGA product was then subjected to a quality control step using the PCR-based Ampli1 QC Kit (Silicon Biosystems). This step was always performed in the designated PCR area, not the area dedicated for single cell WGA. After amplification, 5 μl of QC PCR product was loaded on a 1.2% agarose gel and bands for four PCR products visualized. For those samples with at least one visible band on the Q/C gel, PCR was performed using the Ampli1 ALK, KRAS, or PIK3CA Seq kits (Silicon Biosystems) designed to detect mutations in the DNA libraries obtained using the Ampli1 WGA Kit. In addition, a post-PCR gel was run prior to sequencing to verify amplification of the gene of interest. If no band was detected on the post-PCR gel, sequencing was not conducted. For samples with a positive band on the post-PCR gel, Sanger sequencing was performed to detect known mutations in the *ALK*, *KRAS*, and *PIK3CA* genes. All analyses were conducted using Sequencher (Gene Codes Corporation, Ann Arbor, MI, USA).

### Primer design for sequencing of ALK F1174L mutation

The Ampli1 WGA process depends on the ligation of adaptors to DNA fragments, which are, in turn, generated by genomic digestion with the restriction enzyme at TTAA sequences. Given that the C > A transversion, resulting in the F1174L mutation of *ALK*, results in an extra TTAA enzyme restriction site, our primer design strategy for PCR with the Ampli1 ALK Seq kit had to be altered for accurate detection of this mutation. To address this, separate reactions were conducted to detect the wild-type allele (left intact by restriction enzyme digestion) and the mutant allele. Given that the mutation-containing TTAA sequence would be directly ligated by primers for WGA, this would not leave enough space in the 5’ flanking region for addition of primers for target-specific PCR and accurate detection of the mutation. Therefore, the PCR forward primer was re-designed to overlap the WGA primer ligated directly 5’ of the mutation site. This dictated that all reactions can be run by reverse rather than forward sequencing. Further, the nucleotides directly 5’ of the expected TAA codon will be detected as the adaptor sequence rather than the expected wild-type sequence.

### Short tandem repeat analysis

For cell lines not expressing a known point mutation at the *ALK* locus, tumor cell identity was verified using STR analysis. Genotyping was conducted using an STR-based assay developed by Silicon Biosystems as a multiplex PCR, compatible with Ampli1 WGA digest, with 11 loci detected across 4 fluorescence channels with capillary electrophoresis, using as input only a 1 μl aliquot of the WGA product from single cells, generated as described above. STR reaction mix was added to each sample as well as to a no-amplification negative control. Samples were incubated in a thermal cycler using the following cycles: one 15 min cycle at 94°C and then 32 cycles with each cycle including 50 s at 94°C, 50 s at 60°C, and 1 min at 72°C. The STR call-rate was calculated by dividing the number of alleles detected for each single cell by the number of alleles expected by the STR profile obtained from genomic DNA for the same cell line. No-amplification blanks were used as negative controls. DNA fragment analysis was then conducted on an Applied Biosystems 3730, and the data were analyzed using GeneMarker software (SoftGenetics, State College, PA, USA).

## Results

### Single cell isolation

To first determine whether DEPArray-based cell isolation could be adapted for the capture of tumor cells from patient samples, we conducted 24 experiments using 11 different cell lines, in which 318 tumor cells were collected (Table 1). This includes 208 single tumor cells and 110 tumor cells collected in groups of up to 50 cells. Cell line samples were either composed of all tumor (designated with an “A” in the Spiking ratio column of Table 1) or mixtures of tumor cells and normal donor WBC. For some cell lines, including SY5Y, Kelly, NB1643, and IMR5, tumor cells were first run without mixing with WBC, and subsequent experiments were conducted by titrating indicated amounts of tumor cells with WBC. For all other experiments, either all tumor (CHLA90, SKNAS, and NB1) or spiked tumor/WBC mixtures (LAN5, SW480, H292, and MCF-7) were used as input.

**Table 1:** **Summary of single cells collected from cell line experiments**.

Cell line	Tumor type^a^	Tumor antigen or selection method^b^	Mutation detected	Number of experiments	Spiking ratio^c^	Single tumor cells isolated
SY5Y	N	GD2	ALK (F1174L)	3	A, 1:35, 1:5,000	17
Kelly	N	GD2, CD56	ALK (F1174L)	4	A, A, 1:10, 1:1,000,000	43
NB1643/1643M	N	GD2	ALK (R1275Q)	4	A, 1:10, 1:10, 1:1,000,000	30
LAN5	N	GD2	ALK (R1275Q)	2	1:10, 1:10	21
CHLA90	N	Bright field	ALK (F1245V)	1	A	3
IMR5	N	GD2	–	4	A, A, 1:10, 1:1,000	28
SKNAS	N	Hoechst	–	2	A, A	28
NB1	N	GD2	–	1	A	3
SW480	C	EGFR	KRAS (G12V)	1	1:10	12
H292	L	EpCAM	–	1	1:10	12
MCF-7	B	HER2	PIK3CA (E545K)	1	1:10	11
	
			Totals	24		208

*^a^N, neuroblastoma; C, colon; L, lung; B, breast*.

*^b^GD2, disialoganglioside2; EGFR, epidermal growth factor receptor; EpCAM, epithelial cell adhesion molecule; HER2, human epidermal growth factor receptor 2*.

*^c^Tumor:white blood cell ratio; A, all (100%) tumor*.

**Figure 2:**
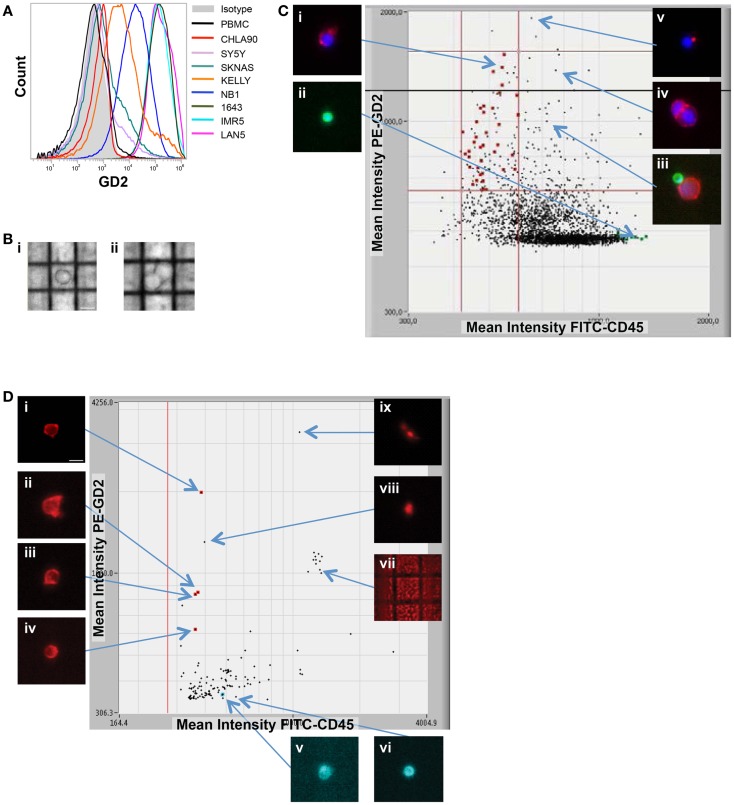
**Image-based capture of individual spiked cells**. **(A)** Cell surface GD2 expression was measured by flow cytometry for the eight neuroblastoma cell lines used for isolation of single cells (and listed at right), as compared to negative control WBCs and isotype control. **(B)** WBCs and the GD2-dim neuroblastoma cell line SY5Y were mixed at a tumor:WBC ratio of 1:35, pre-labeled with GD2-PE, CD45-FITC, and Hoechst nuclear dye, then injected into the DEPArray. Shown are bright-field images of (i) a single tumor cell (bar depicts 10 μm) and (ii) a cluster of two cells. In **(C)**, a scatter plot of GD2-PE and CD45-FITC mean fluorescence intensity (MFI) is shown as well as cell images, including on the left-hand side of the dot-plot (i) an image of a single tumor cell and (ii) a single WBC, and on the right-hand side (iii) a heterogeneous cluster, (iv) a homogeneous cluster, and (v) a spurious event. **(D)** NB1643M cells and normal donor WBCs were mixed at a ratio of 1:1,000,000 and stained with GD2-PE and CD45-FITC, in this representative experiment. The sample was pre-enriched using FACSAria-based sorting, and the enriched fraction placed into the DEPArray cartridge. Shown are a scatter plot of GD2-PE and CD45-FITC MFI, as well as images of (i–iv) intact tumor cells, (v and vi) WBCs, and (vii–ix) debris and false-positive events.

Here, we focus on the pediatric cancer neuroblastoma, a malignancy of neuro-endocrine origin, which does not express the epithelial marker EpCAM (33). Therefore, we first sought to validate cell surface tumor antigens with which tumor cells could be specifically isolated from patient bone marrow samples. Both the disialoganglioside GD2 and the adhesion molecule CD56 have been shown to be widely and brightly expressed on neuroblastoma tumors (33, 34), however, given that CD56 is also expressed at varying cell surface density on subsets of natural killer cells (35), all experiments were carried out using GD2 for positive selection, and CD45 for negative selection of cells of hematopoietic origin. In some experiments, cells were also labeled for CD56 and antigen expression visualized for single tumor cells also expressing GD2. As detected by flow cytometry, the eight neuroblastoma cell lines assayed had a wide range of GD2 expression (Figure 2A). While there was no detectable antigen on the cell surface of CHLA90 and SKNAS, some cell lines, such as SY5Y, had overall dimmer expression and a subset of cells that were negative for GD2, compared to cell lines such as IMR5, which were uniformly bright and positive for this cell surface molecule. Despite this range in cell line tumor antigen expression, single tumor cells were successfully detected and isolated in cell line spiking experiments for all six GD2-positive neuroblastoma cell lines, and over a 5-log range of tumor:WBC ratios (Table 1).

While some experiments were carried out using live cells as input, some samples were either fixed or frozen prior to processing on the DEPArray. The intention here was to mimic the conditions that might be necessary for the preservation of a clinical sample if immediate processing was not possible. Tumor/WBC mixtures were labeled with antibodies to GD2, CD45, and, in some cases, CD56, and an aliquot was injected into the DEPArray. Cells were then selected based on bright-field images, allowing for discrimination of single versus clusters of cells (Figure 2B), and based on dot plots of mean fluorescence intensity (MFI), much the same as the output from a flow cytometer (Figure 2C). However, unlike with flow cytometry, on the DEPArray images can be immediately visualized for each event (or dot), thus allowing for on-chip, image-based phenotypic and morphologic assessment of each cell if desired, as well as further discrimination of doublets, non-specific binding of antibody conjugates, and other spurious events that would likely have appeared as a single tumor cell by flow cytometry. Representative single cell images are shown in Figure 2C for a mixing experiment in which the GD2-dim cell line SY5Y was mixed with WBC at a ratio of 1:35. To assess whether single tumor cells could be isolated by means other than cell surface staining, the GD2-negative cell lines CHLA90 and SKNAS were isolated by bright-field (Figure 2B) and Hoechst nuclear dye, respectively. To determine whether our approach could be extended to other histotypes and cell surface markers, we also conducted spiking experiments with the breast cancer cell line MCF-7 (isolated based on HER2 labeling), colon cancer line SW480 (EpCAM), and the non-small cell lung cancer line H292 (EGFR) (experimental parameters listed in Table 1). These results suggest a robust and adaptable approach for single cell isolation.

### Capture of very rare single cells

Given that neuroblastoma patient blood and bone marrow samples have been shown to have a wide range of tumor involvement (24), we next wanted to establish the sensitivity of the DEPArray for isolation of extremely rare tumor cells. To do this, we used cell line spiking experiments at concentrations of 1 tumor cell in 10^6^ WBCs for two different cell lines, Kelly and NB1643M (a sub-clone of the NB1643 cell line, which is homozygous for the R1275Q mutation in the *ALK* gene). For the representative experiment shown in Figure 2D, serial dilutions were used to generate a sample with 10 NB1643M tumor cells in 10 million WBCs. Cells were fluorescently labeled as described above and flow cytometry-based sorting of the entire cell population used to pre-enrich the sample prior to loading into the DEPArray cartridge. As discussed above in the Materials and Methods section, this was necessary for any sample with less than a 0.02% tumor cell concentration because of the 40,000 cell capacity of the cartridge. In this case, for a sample with a tumor cell concentration of 1 in 10^6^ WBC, the probability of having even 1 tumor cell among all cells loaded into a cartridge with a 40,000 cell capacity would be very low, and loading the entire 10 million cells onto multiple cartridges would have been unfeasible. Therefore, flow cytometry-based sorting was utilized to enrich the tumor cell population prior to loading the sample on the DEPArray. Cells were flow sorted using a sufficiently large enrichment gate to ensure maximal capture of GD2-positive/CD45-negative cells. This enriched fraction was then either run immediately on the DEPArray or fixed cells were stored for up to 5 days before purification of single cells on the DEPArray. Especially for the capture of such rare cells, on-chip single cell imaging was essential for distinguishing the four intact GD2-positive/CD45-negative single cells from high MFI debris or non-specifically stained material (dot-plot and representative single cell images shown in Figure 2D). This capture rate of 40% (4 cells out of the original 10 spiked) is commensurate with cytometry- (36, 37) and dielectrophoretic-based capture (38) of tumor cells spiked at a ratio of 1 tumor cell in one million WBCs. Given that an average capture rate of 60% of input cells has already been demonstrated for DEPArray (19), yields were not calculated for the rest of the experiments described below. Taken together, these data suggest a highly specific and sensitive approach for the isolation of very rare single tumor cells.

### Single cell WGA and targeted sequencing

**Figure 3:**
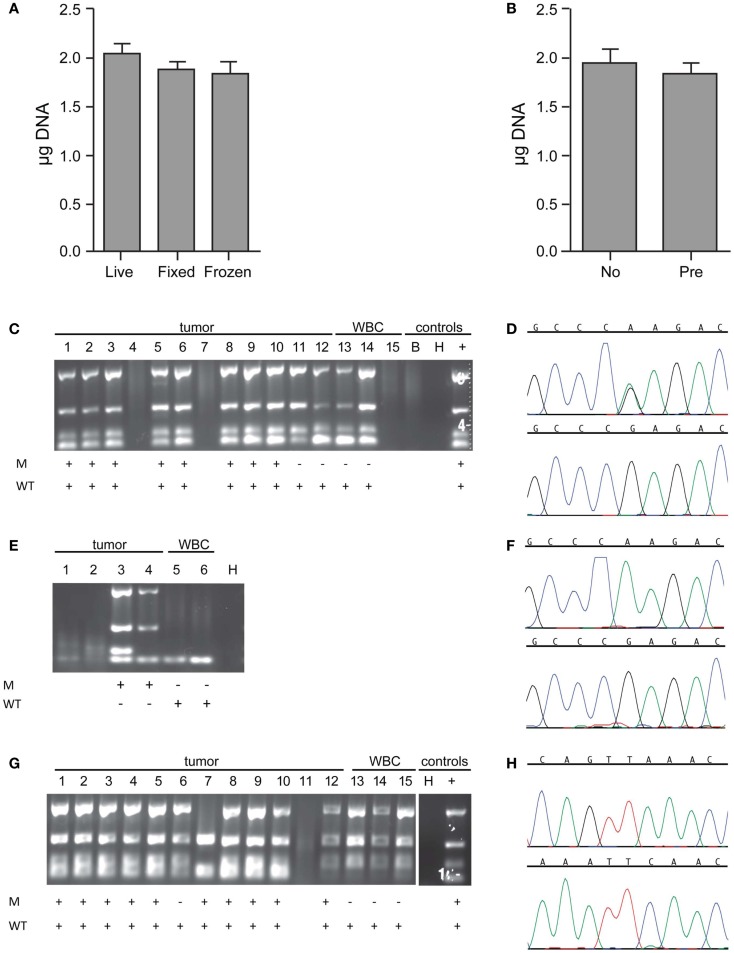
**Whole genome amplification and targeted sequencing of single cells**. Single cells were isolated by DEPArray, DNA extracted, Ampli1 WGA performed, and the DNA concentration of each sample measured by Picogreen. **(A)** The mean WGA yield in micrograms of DNA for the 50 μl elution volume for *n* = 58 live (unfixed) single cells, *n* = 95 fixed single cells, and *n* = 26 frozen/thawed single cells; and **(B)** *n* = 40 single cells from unenriched samples (left bar) and *n* = 55 single cells (right bar) from samples that had been pre-enriched by flow sorting prior to single cell capture on the DEPArray. Included in this analysis are single cells isolated from spiked cell lines as well as patient samples described in the text. For the data shown in **(B)**, all cells were fixed. **(C)** A 1:10 mix of NB1643 cells and WBCs was pre-labeled, single cells collected by DEPArray, DNA extracted, and WGA performed. WGA product was subjected to a quality control step in which bands for 4 housekeeping genes were visualized, as shown for 12 single heterozygous NB1643 tumor cells (columns 1–12), 3 WBCs (columns 13–15), a no-cell “blank” (B), a water control (H), and positive control genomic NB1643 DNA (+). Detection of the mutant allele is denoted by “+” in row M, and detection of the wild-type allele in Row WT. **(D)** WGA products with one or more bands were submitted for targeted sequencing at the *ALK* locus. Shown are chromatograms depicting the G > A transition (R1275Q) for NB1643 cell #3 (top) and wild-type sequence for WBC#13 (below). **(E)** NB1643M tumor cells, homozygous for the R1275Q mutation, were spiked into WBCs at a 1:1,000,000 ratio, and single cells were captured. Shown is the Q/C blot for the four tumor cells and two WBCs isolated (corresponding dot-plot and single cell images shown in Figure 2D), with sequencing results below the blot and **(F)** the chromatogram for tumor cell #3 depicting the known homozygous G > A transition (top) and for WBC#6 showing wild-type sequence (bottom). **(G)** A 1:10 mix of Kelly cells and WBCs was frozen at -80°C, then thawed and stained before DEPArray-based capture and WGA. Shown is the Q/C gel for 12 single Kelly cells, 3 WBCs, a water control (H), and genomic Kelly DNA (+). Detection of the mutant and wild-type alleles is depicted below. **(H)** Chromatograms for tumor cell #2 depicting the mutant allele (top panel) and the wild-type allele (bottom panel). As described in the Materials and Methods section, for cell lines such as Kelly expressing the heterozygous F1174L mutation, sequencing of the mutant and wild-type alleles are conducted in separate reactions.

To generate sufficient starting material for genetic analysis of single cells, Ampli1 WGA was performed. For the 179 single cell samples, concentration of WGA product was measured by direct fluorescent staining (including single cells isolated from cell lines as well as the patient samples described in more detail below), and the mean WGA product concentration was 38.44 ng/μl in a 50 μl volume for a total WGA product of 1.922 μg (± 0.0610 μg, range 0.243–4.048 g) per sample. To assess the effect of sample preparation on the amount of WGA product, we compared results for live (*n* = 58), fixed (*n* = 95), or frozen/thawed (frozen after capture, stored at -80°C, then thawed prior to WGA; *n* = 26) cells, and found no difference in yield (*p* = 0.1530 for live versus fixed, *p* = 0.1548 for live versus frozen/thawed, *p* = 0.3756 for fixed versus frozen/thawed; Figure 3A). Similarly, and comparing among fixed samples only, whether the sample was pre-enriched by FACS (*n* = 55) or not (*n* = 40) had no effect on WGA yield (*p* = 0.2890; Figure 3B). To assess WGA product quality, PCR for four control sequences was performed. As shown in Figure 3C for a spiking experiment using live NB1643 cells, four bands were detected on the Q/C gel for 12 of the 15 single cells. No bands were detected in this or any other experiment for the no-cell blank collected from the DEPArray cartridge. Only cells with one or more visible bands were selected for further analysis, since the absence of any bands suggests that either the cell was lost subsequent to DEPArray capture or the WGA reaction incomplete. To verify tumor versus WBC identity, WGA products for the 12 cells with visible bands were amplified using primers specific for the known heterozygous R1275Q *ALK* mutation, and targeted sequencing performed. As shown in the chromatograms in Figure 3D, the expected mutation (top) and wild-type sequence (bottom) were detected in a tumor cell and WBC, respectively. Targeted sequencing results for all cells are shown below the gel in Figure 3C, and indicate that the expected mutant allele was not detected in tumor cells #11 and 12. This could be due to cell to cell heterogeneity, allelic dropout (ADO), or a WBC having been isolated rather than a tumor cell. No mutant allele was detected in either of the WBCs sequenced here, or for the 161 WBCs isolated across all experiments described in this report. To determine whether this approach could be used for genetic analysis of very rare cells, we analyzed single cells captured subsequent to FACS-based pre-enrichment of a sample with tumor cell concentration of 1:1,000,000 (cell images and dot-plot shown in Figure 2D). For this experiment, the NB1643M cell line expressing a homozygous ALK mutation was used, and Q/C analysis of the WGA products resulted in one or more bands for four of the six captured cells (two tumor and two WBCs; Figure 3E). Sequencing results shown in Figure 3E indicate detection of the expected mutation in the two tumor cells and wild-type sequence in the two WBCs (representative chromatograms in Figure 3F).

To further explore the effect of freezing/thawing cells, we conducted experiments in which cell lines were spiked into WBCs, half the mixture stained and immediately run on the DEPArray, whole genome amplified and sequenced, and the other half frozen at -80°C for several weeks before further processing. As shown on the representative Q/C blot for the Kelly neuroblastoma cell line in Figure 3G, one or more bands were detected on the Q/C gel for 14 out of 15 single cells, the mutant allele for this heterozygous cell line detected in 10 out of 11 tumor cells, and the wild-type allele detected in all cells sequenced. Representative chromatograms shown in Figure 3H depict the known F1174L mutant allele (top) and the wild-type allele (bottom) for tumor cell #2 run in separate reactions (as described in the Materials and Methods section). Moreover, there was no evidence of antigen degradation or change in staining pattern when comparing fresh with frozen/thawed single cell images (representative single cells shown in Figure A1 in Supplementary Material).

Verification of tumor cell identity for single cells isolated by DEPArray from spiking experiments using the SW480 colon carcinoma cell line and the MCF-7 breast cancer cell line was achieved by sequencing of the known KRAS (G12V) and PIK3CA (E545K) mutations, respectively (Table 1). For neuroblastoma cell lines not known to harbor an ALK mutation, we used genotyping of short tandem repeats (STRs) at 11 loci. As shown for two loci in Figure A2 in Supplementary Material, the genotype of single tumor cells was consistent with that of unamplified genomic DNA from the same cell line but distinct from the WBC isolated from the same spiking experiment, thus demonstrating an additional means of validating tumor cell identity.

**Figure 4:**
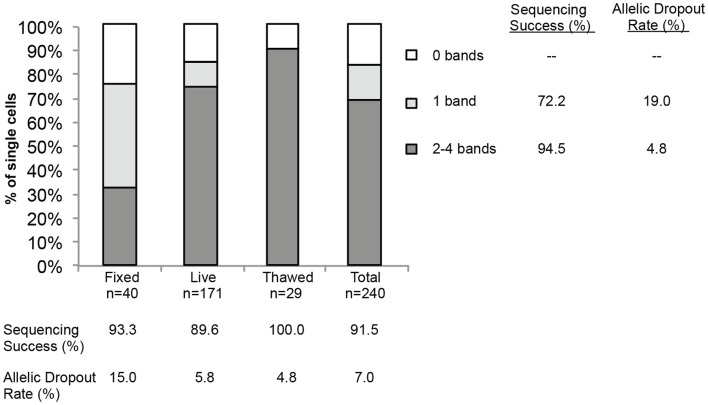
**Sequencing success and allelic dropout (ADO) rate**. Two hundred forty single cells, including tumor cells (*n* = 208) and WBCs (*n* = 32), were collected from spiking experiments and subjected to WGA. Among the 240 single cells, 40 fixed, 171 live, and 29 thawed cells were analyzed for the number of bands detected on the post-WGA Q/C gel. The height of each bar represents the percentage of cells with 2–4 (dark grey), 1 (light grey), or 0 bands (white) on the Q/C gel. Shown at right and below the graph are the sequencing success rates for all 240 tumor cells and WBCs by category, as well as the allelic dropout rate for the 110 tumor cells sequenced.

Finally, to more comprehensively assess the effect of cell preparation on sequencing reliability, we compared results for 240 single cells (208 cell line and 32 WBCs), including fixed (*n* = 40), live (*n* = 171), or freeze/thawed (*n* = 29) cells (Figure 4). Sequencing was conducted and ADO rate was calculated only for cells for which one or more bands were detected on the Q/C gel. Overall sequencing success, defined as the percentage of single cell WGA products for which a sequence result was obtained, was 91.5%, and ranged from 89.6% for live, 93.3% for fixed, and 100.0% for thawed cells. The ADO rate for tumor cells (the percentage of known alleles not detected) was 7.0%, with live and thawed cells having dropout rates of 5.8 and 4.8%, respectively. Fixed cells had a higher ADO at 15.0%, and an even higher proportion (42.5%) of cells with only one band detected on the Q/C gel. Given the likely fixation-induced DNA cross-linking, these results are not unexpected. In fact, three of the four Q/C PCR targets are located in very large amplicons (>1100 base-pairs, above 99th percentile of the size distribution of WGA digest) and are more difficult to amplify. The ADO rate for smaller post-WGA fragments (comprising most of the WGA library at 100–200 base-pairs) is typically lower. To determine the relationship between number of Q/C bands and sequencing success and ADO, we tabulated Q/C results by cell preparation method (Figure 4). Although the overall percentage of single cells (tumor and WBCs) with 2–4 Q/C bands varies widely depending on cell preparation (fixed 32.5%, live 73.7%, thawed 89.6%, and overall 68.8%), the percentage with one or more varied less (fixed 75.0%, live 84.2%, thawed 89.6%, and overall 83.3%). Cells with 2–4 bands had a sequencing success rate of 94.5%, and an ADO of 4.8%, whereas cells with 1 band had lesser results, with sequencing success of 72.2% and ADO of 19.0%. These results suggest that although sequencing reliability and accuracy are higher for cells with 2–4 bands on the Q/C gel, the vast majority of cells with at least 1 Q/C band will generate accurate sequence data. Taken together, these results demonstrate a reliable approach for the WGA and sequencing of single tumor cells, and that multiple approaches can be used for preserving samples that cannot be immediately processed.

### Capture and genetic analysis of single cells from patient samples

**Figure 5:**
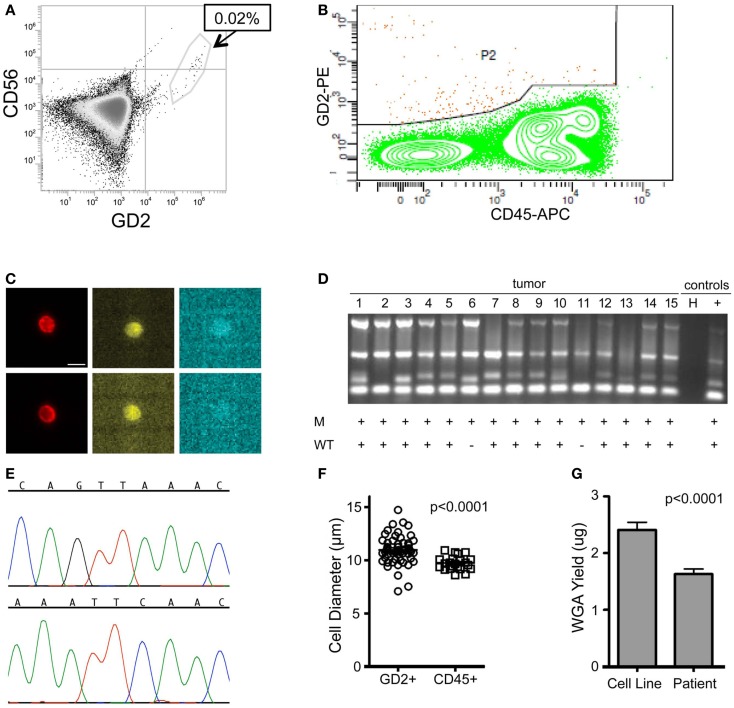
**Isolation and targeted sequencing of rare individual patient DTC**. **(A)** The post-Ficoll fraction for a bone marrow sample from patient CHOP7 was fixed, stained for CD45, CD56, and GD2, and an aliquot was analyzed by flow cytometry. Given that only 0.02% of cells were determined to be GD2-positive, **(B)** pre-enrichment was conducted on the FACSAria using the wide P2 gate shown. **(C)** Representative images of staining for GD2 (first column; bar depicts 10 μm), CD56 (middle column), and CD45 (right column) are shown for single cells #15 (top row) and #7 (bottom row). **(D)** A gel for the quality control panel of four housekeeping genes is shown with a “+” below depicting detection of either the mutant allele (top row labeled “M” or the wild-type allele bottom row labeled “WT”). **(E)** The chromatogram of the wild-type (top) and mutant allele (bottom) for cell #8. **(F)** Diameter of patient cells, as measured for 56 tumor cells and 22 WBCs while in solution on the DEPArray chip. **(G)** Bone marrow samples of patients were processed as described above and direct fluorescent staining used to measure the DNA concentration of WGA product from single cells. Mean WGA yield for *n* = 64 patient single cells is shown in comparison to the yield for *n* = 31 single tumor cells from cell line spiking experiments. In this analysis, all patient and cell line cells were fixed.

To assess the feasibility of clinical application of single tumor cell capture and genetic analysis, we next applied our approach to 10 bone marrow samples of patients. As outlined in Table 2, this includes seven samples with known ALK status in the corresponding primary tumor (as detected by our Molecular Genetics Laboratory), and three samples for which ALK status of the primary tumor was unknown. As also detailed in Table 2, for patient 9, two different bone marrow samples were analyzed and are listed as samples 9a and 9b. The post-Ficoll fractions of all patient samples were fixed before processing, and all together, 178 total single cells (144 DTCs and 34 WBCs) were collected from these 10 bone marrow samples of patient. To assess the level of tumor involvement prior to running the sample on the DEPArray, each sample was stained for GD2, CD56, and CD45, and an aliquot of cells run on the flow cytometer (see FACS results for representative patient CHOP7 in Figure 5A). For this and all other patients with a percentage of GD2-positive tumor cells at or below our predetermined threshold of 0.02%, we next conducted pre-enrichment on the FACSAria by setting a wide collection gate (depicted by P2 in Figure 5B), and then injected the enriched product into the DEPArray cartridge and collected 15 single tumor cells (two representative images shown in Figure 5C; no WBCs were collected in this experiment). Consistent with the flow cytometry results in Figure 5A, single cell images of all tumor cells collected showed brightly expressed GD2 but there was heterogeneity of CD56 expression with some cells more strongly expressing CD56 (Figure 5C, top row) and others expressing the antigen more dimly (Figure 5C, bottom row). All isolated tumor cells had one or more bands on the Q/C gel and were, therefore, sequenced to verify presence of the heterozygous F1174L mutation that had been detected in the patient’s primary tumor. Indeed, the mutant allele was detected in all 15 cells and the wild-type allele in 13 out of 15 cells, implying that two tumor cells were either homozygous for the mutant allele or had suffered from ADO of the wild-type allele during WGA (Figure 5D). Shown in Figure 5E are the chromatograms for tumor cell #8 including the wild-type allele (top) and the mutant allele (bottom) run in separate reactions as described above. These results demonstrate concordance between the targeted sequencing results for the solid tumor and DTCs from patient CHOP7. Despite the wide range in tumor involvement for all patients studied (0.01–80.0%; Table 2), similar concordance was shown for all patients with known primary tumor ALK status.

**Table 2:** **Summary of single DTCs collected from neuroblastoma patient bone marrow samples**.

Patient	FACS-based pre-enrichment	Bone marrow % tumor^a^	Primary tumor ALK status	DTC ALK status	Single tumor cells isolated	WBC isolated
CHOP1	No	0.12	Unknown	–	5	4
CHOP2	No	19.00	Unknown	–	6	10
CHOP4	No	0.11	Unknown	–	9	0
CHOP6	Yes	0.01	WT	WT	15	0
CHOP7	Yes	0.02	F1174L	F1174L	15	0
CHOP8	No	4.00	F1174L	F1174L	22	3
CHOP9a	Yes	N/A	Y1278S	Y1278S	12	2
CHOP9b	No	80.00	Y1278S	Y1278S	12	3
CHOP10	No	1.4	WT	WT	24	6
CHOP11	No	40.00	WT	WT	24	6
	
				Totals	144	34

*^a^As measured by flow cytometry*.

Overall, we found that patient marrow samples ran without complication on the DEPArray apparatus. Based on analysis of the diameter of patient single cells recovered by DEPArray, we determined that there was considerable overlap between the 56 GD2+ tumor cells and 22 WBCs measured (Figure 5F), suggesting that commonly used cell size-based filtration methods would be unlikely to provide sufficient pre-enrichment of disseminated neuroblastoma tumor cells. As previously discussed (and displayed in Figures 4A,B), the overall mean WGA yield for 95 single cells that had been fixed prior to WGA was 1.884 + 0.8145 μg. However, that yield varied considerably between WGA products for single cells from cell lines and patient samples, with the WGA yield for 64 patient single cells (1.629 + 0.09011 μg; all cells fixed) being significantly lower than that seen for 31 fixed single cells from cell line experiments (2.409 + 0.1349 μg, *p* < 0.0001; Figure 5G). Nevertheless, the lower yield from patient cells was still sufficient for multiple downstream analyses. When aliquots of WGA product were run on a Q/C gel, one or more bands were detected in 68.5% of patient single cells, which was slightly below the 75.0% achieved for fixed single cells from cell line spiking experiments. For the cells with one or more Q/C bands, a sequencing result was obtained for 87.7% of patient and 93.3% of fixed spiked cell line cells. ADO rates were also similar with patient cells having a slightly lower rate of 12.7% as compared to spiked cell line cells at 15.0% (Table 3). These data suggest a robust and highly sensitive approach for capture of single tumor cells from neuroblastoma patient samples, and detection of known point mutations.

**Table 3:** **WGA and sequencing of fixed single cells**.

Type of sample	Cells with ≥ 1 Q/C band^a^ (%)	Cells successfully sequenced^a^ (%)	Allelic dropout^b^ (%)
Patient	68.5	87.7	12.7
Cell line	75.0	93.3	15.0

*^a^Tumor cells and WBCs*.

*^b^Tumor cells only*.

## Discussion

We have presented here a reliable and adaptable approach for the capture and downstream analysis of single tumor cells. Routine sampling and interrogation of the circulating and disseminated tumor compartments facilitate important basic science and translational medicine goals. In order to realize the true benefits of personalized cancer therapy, molecular profiling of patient tumors must extend beyond limited sampling of bulk primary tumor tissue. Repeated invasive procedures, however, are not an acceptable means of addressing this need and, for many cancer patients with advanced disease, access to the primary tumor is impossible. Indeed, for children suffering from neuroblastoma, the cancer focused on in this study, primary tumor samples are often difficult or impossible to obtain, due to the tumor’s paraspinal and/or bony location. Curative therapies have yet to be identified in the relapse setting for neuroblastoma patients, underscoring the need for new approaches to molecular characterization of the tumor that will lead to more effective therapeutic strategies. Limited access to primary tumor makes it challenging to ascertain the status of molecular markers such as point mutations in *ALK*. While the most common ALK variants (R1275Q and F1174L) in neuroblastoma confer differential sensitivity to the kinase inhibitor crizotinib (39), a compound with promising results in a recent Phase I trial (31), identifying candidate patients for this targeted therapy and determining their mutation status is often impossible when primary tumor tissue is unavailable. Here, we have shown an approach for the capture of very rare cells from starting populations as low as 1 in one million cells. Using this approach, we were able to isolate DTCs from marrow samples with a tumor cell concentration as low as 0.01%, and demonstrate concordance for *ALK* status between primary tumor and DTCs for all patients for whom status of the primary tumor had been ascertained. This suggests an approach that can be adapted to provide crucial clinically relevant information for the stratification of neuroblastoma patients onto therapy with an ALK inhibitor.

While several groups have demonstrated the feasibility of accessing and analyzing circulating tumor (3, 6, 40, 41), current approaches are largely limited by their reliance on EpCAM as a cell surface marker with which to differentiate tumor from WBCs in the blood or marrow. More recently developed approaches have incorporated additional markers for CTC/DTC detection, including markers for the detection of tumor cells that have undergone EMT, and yielding improved sensitivity and the detection of EpCAM-negative subsets of CTC/DTCs (6, 40, 42). For example, multiparametric flow-based sorting, including labeling of multiple cell surface markers on breast cancer CTCs, led to the identification of a possible signature of brain metastasis among EpCAM-negative cells (40). In addition, Yu and colleagues recently used labeling with multiple antibodies and a microfluidic approach to isolate circulating breast tumor cells from patient blood and showed considerable variability in epithelial and mesenchymal characteristics across different histological subtypes of breast cancer (6). However, it has also become increasingly clear that human cancers demonstrate complex subclonal heterogeneity, suggesting that CTC/DTCs will also be heterogeneous in terms of potentially targetable genetic alterations. Using current approaches to isolate CTC/DTCs, enriched cells cannot be individually isolated for downstream genetic analysis. The results we report here demonstrate proof of principle that individual tumor cells can now be captured for downstream genetic analysis.

Although use of the DEPArray for CTC isolation from patient blood samples has been reported (18, 19), the work described here provides a comprehensive method for microfluidic-based isolation and genetic analysis of single DTCs from patient marrow samples. DTCs from neuroblastoma patient samples were isolated based on expression of the cell surface marker GD2, while spiked cell line tumor cells were detected and captured by labeling for GD2, HER2 (breast), EGFR (NSCLC), and EpCAM (colon). In an effort to extend our approach to additional histotypes, we are currently adapting the above described DTC-isolation protocols for breast cancer patient marrow samples, and work is ongoing to apply GD2-based isolation approaches to the blood samples of patients with melanoma and small cell lung cancer (SCLC) as both tumors have been shown to express this cell surface marker (43, 44). We have also demonstrated the feasibility of both fixation and freezing as approaches to temporally disassociate the acquisition and processing of patient samples, a necessary component for the routine analysis of clinical samples. We are currently working to enhance the sensitivity of detecting CTC/DTCs of epithelial origin before and after EMT by adding epithelial markers such as EpCAM and E-Cadherin and mesenchymal markers including vimentin and N-Cadherin. Use of multiple markers will enhance the yield of captured CTC/DTCs from among a heterogeneous population, with the goal of eventually extending this platform to disease monitoring and the detection of minimal residual disease, especially for patients in radiographic remission. Importantly, such adaptation is easily achievable with the DEPArray as the combination of staining antibodies can be adjusted for each sample or histotype without the re-fabrication or re-coating of the internal structure of the chip.

Until recently, the downstream analysis of patient samples has largely focused on endpoints that technology could afford, namely genomic profiling of homogenized primary tumor samples with little regard to intratumoral heterogeneity. Major technological challenges have included the reliable amplification and analysis of single cell DNAs, with the 6–7 pg of genomic DNA typically extracted from a single cell (45) being insufficient for most downstream analysis. Introduction of WGA artifacts and DNA contamination during single cell DNA amplification have also impeded progress. These challenges were addressed in recent reports in which single cell sequencing was shown to provide unique insights into a limited number of cancer genomes (46–48). Here, we provide comprehensive quantification of the accuracy and success rate of each step in a detailed approach for genetic analysis of single tumor cells. To generate sufficient DNA for targeted sequencing of isolated single cells, we used Ampli1 global amplification based on restriction enzyme digest, adaptor ligation, and PCR amplification. Among cell lines, WGA and targeted sequencing were conducted at five loci [KRAS(G12V), PIK3CA(E545K), and ALK(F1174L, R1275Q, and F1245V)], suggesting that this approach could be extended to the detection of point mutations in multiple genes, including those associated with resistance to kinase inhibitors and other targeted therapies. Indeed, the F1174L point mutation detected here in neuroblastoma patients prior to therapy has also been identified as a resistance mechanism in a patient with an inflammatory myofibroblastic tumor who was treated with Crizotinib (49).The fidelity of the WGA process as measured by ADO rate was 5.8% for live single cells, a rate that is commensurate with recently published studies (46, 50). Although the ADO for patient samples was higher at 12.7%, all patient samples were fixed, and this rate for patient samples is comparable to the 15.0% ADO measured for spiked fixed cells. Importantly, these ADO rates were calculated only for cells for which one or more bands were detected on the Q/C gel of four housekeeping genes. No mutant allele was detected in any of the 161 WBC sequenced, suggesting a lack of cross-contamination between samples. Sequencing of single cells isolated from the bone marrow of patients whose primary tumors harbored a heterozygous ALK mutation sometimes resulted in either a homozygous wild-type or homozygous mutant call. Although this suggests the possibility of cell to cell heterogeneity at this locus, more samples would have to be studied to determine whether this is true heterogeneity. While unlikely, a homozygous wild-type call could have resulted from isolation of extremely rare GD2-positive CD45-negative bone marrow mesenchymal stem cells (51). Finally, both false-negative and false-positive calls may simply be reflective of the underlying ADO rate of the WGA process. Nevertheless, reductions in ADO rate resulting from rapid advancement in single cell genomic technology (46, 47, 52), coupled with DEPArray-based tumor cell isolation promise the routine and accurate detection of clinically relevant and therapeutically targetable genetic aberrations in a non-invasive manor. As efforts continue to streamline and shorten processing times for single cell isolation and WGA, performing such tests in a clinical setting will become more achievable.

Here, we have used neuroblastoma as a model for the study of disseminated tumor cells. Our development of a pipeline for sequence level analysis of individual disseminated metastatic cancer cells isolated from patient samples is but the first step in realizing the basic science and clinical benefits of studying this compartment. Ultimately, a comprehensive genome-wide understanding of the genetic markers and drivers of metastasis will require next generation, rather than targeted, sequencing of single or few cells. Development of clinical tests will likely require simultaneous interrogation of a panel of genes in order to be truly therapeutically meaningful. Addressing these challenges and developing both research- and clinical-lab approaches to the study of CTC/DTCs will lead to important advancements in the treatment of cancer.

## Conflict of Interest Statement

The authors declare that the research was conducted in the absence of any commercial or financial relationships that could be construed as a potential conflict of interest.

## Supplementary Material

The Supplementary Material for this article can be found online at http://www.frontiersin.org/Journal/10.3389/fonc.2014.00201/abstract
