# Supplementary material for: Dielectrophoretic Capture and Genetic Analysis of Single Neuroblastoma Tumor Cells
Source: Front Oncol. 2014 Jul 31;4:201. doi: 10.3389/fonc.2014.00201 (PMC4116800; doi:10.3389/fonc.2014.00201)
Supplement: Supplementary file 1 [file Presentation_1.PDF]

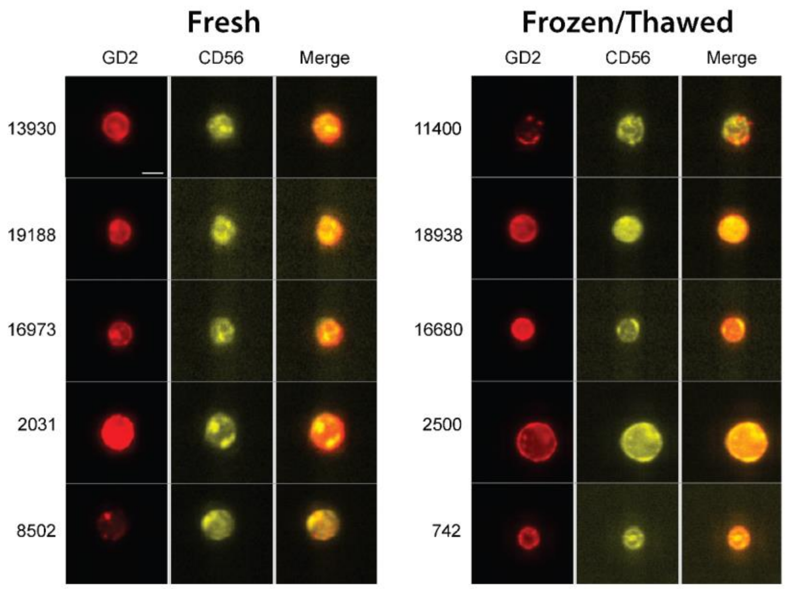

Figure A1. Antigen visualization pre-and post-freeze/thaw. Kelly neuroblastoma cells were spiked into WBCs at a 1:10 ratio and divided into two aliquots. Half the cells were immediately stained and run on the DEPArray and single cells collected, while the second half was frozen at -80C for several weeks and then thawed prior to staining and single cell collection. As shown for each of 5 fresh (left panel) and frozen/thawed (right panel) single cells, expression of GD2 (first column; bar depicts 10μm) and CD56 (middle column) were unaffected in these representative images. Numbers to left of each image are DEPArray-assigned cell ID numbers.

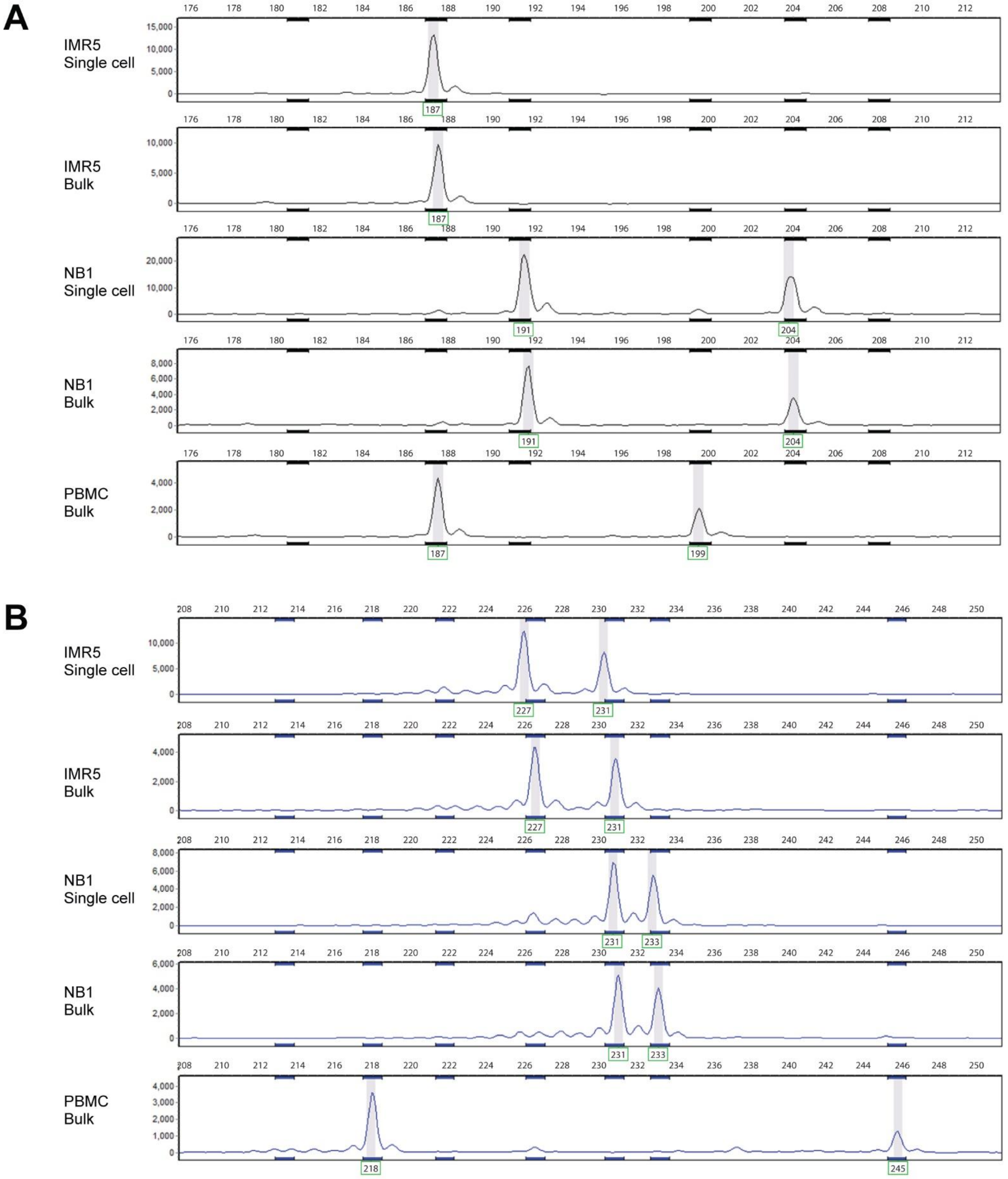

Figure A2. Verification of tumor identity by STR analysis. Detection of STRs was used to verify the identity of spiked neuroblastoma tumor cells expressing wild-type ALK. Genotyping at 11 loci was conducted using the Ampli1 STR Kit. Representative results for the D13S317 (A) and D21S11 (B) loci are shown comparing single cell WGA product and unamplified bulk IMR5 neuroblastoma cells (first and second rows), NB1 neuroblastoma cells (third and fourth rows), and WBC (unamplified bulk; fifth row).
